# The monoamine stabilizer (−)‐OSU6162 prevents the alcohol deprivation effect and improves motor impulsive behavior in rats

**DOI:** 10.1111/adb.12613

**Published:** 2018-02-26

**Authors:** Ida Fredriksson, Malin Wirf, Pia Steensland

**Affiliations:** ^1^ Department of Clinical Neuroscience, Karolinska Institutet Karolinska University Hospital Sweden

**Keywords:** alcohol use disorder, dopamine stabilizer, five‐choice serial reaction time task, impulse control, relapse‐like drinking

## Abstract

Alcohol craving, in combination with impaired impulse control, often leads to relapse. The dopamine system mediates the rewarding properties of alcohol but is also involved in regulating impulsive behavior. The monoamine stabilizer (−)‐OSU6162 (OSU6162) has the ability to stabilize dopamine activity depending on the prevailing dopaminergic tone and may therefore normalize the dopaminergic transmission regulating both alcohol use disorder and impulsivity. We have recently showed that OSU6162 attenuates voluntary alcohol consumption, operant alcohol self‐administration, alcohol withdrawal symptoms and cue‐induced reinstatement of alcohol seeking in rats. Here, we evaluated OSU6162's effects on motor impulsivity in Wistar rats that had voluntarily consumed alcohol or water for 10 weeks. The five‐choice serial reaction time task was used to measure motor impulsivity, and a prolonged waiting period (changed from 5 to 7 seconds) was applied to induce premature responses. OSU6162‐testing was conducted twice a week (Tuesdays and Fridays), every other week with regular baseline training sessions in between. We also tested OSU6162's effects on the alcohol deprivation effect in long‐term alcohol drinking Wistar rats. The results showed that OSU6162 (30 mg/kg) pre‐treatment significantly improved motor impulsivity in the five‐choice serial reaction time task in both alcohol and alcohol‐naïve rats. Moreover, OSU6162 (30 mg/kg) pre‐treatment prevented the alcohol deprivation effect, i.e. relapse‐like drinking behavior after a forced period of abstinence in long‐term drinking rats. In conclusion, our results provide further support for OSU6162 as a novel treatment for alcohol use disorder. The results further indicate that improvement of motor impulse control might be one mechanism behind OSU6162's ability to attenuate alcohol‐mediated behaviors.

## Introduction

A main problem in the treatment of alcohol use disorder (AUD) is the long‐lasting vulnerability to relapse (O'Brien 2005). Alcohol craving commonly precedes relapse and can be triggered by stress (Sinha *et al*. 2011), acute exposure to the drug (i.e. priming) (de wit 1996) or drug‐associated cues (O'Brien *et al*. 1992). Impaired impulsive control, often seen in AUD individuals (Lejuez *et al*. 2010), might further contribute to relapse to alcohol drinking (Bowden‐Jones *et al*. 2005). In fact, clinical studies have found detoxified alcohol dependent individuals to demonstrate poor inhibitory control in several behavior tasks, such as stop signal serial reaction (Lawrence *et al*. 2009), continuous performance task (Bjork *et al*. 2004) and Go/No‐Go task (Kamarajan *et al*. 2005). Thus, it has been suggested that improvement of impulsive control might provide an effective treatment approach to prevent relapse.

Activation of the mesolimbic dopamine system contributes to the acute reinforcing and rewarding effects of alcohol (Vengeliene *et al*. 2008). In contrast, chronic alcohol consumption reduces the number of D2 receptors (Volkow *et al*. 2002) and decreases dopamine release (Volkow *et al*. 2007). This dopamine deficiency has been hypothesized to contribute to alcohol craving and relapse to drinking even after a long period of abstinence (Guardia *et al*. 2000). Several pre‐clinical and clinical studies indicate that dopamine, in distinct brain regions, regulates also impulsive behavior (Dalley & Roiser 2012). For example, amphetamine‐induced enhancement of dopamine activity in the nucleus accumbens (NAcc) increases impulsive behavior in rodents (Cole & Robbins 1987). An effect that can be counteracted by a selective D2 receptor antagonist (Pattij *et al*. 2007). Nevertheless, dopamine hypoactivity in prefrontal regions (anterior cingulate/ventromedial cortex) might also increase impulsive behavior (Fineberg *et al*. 2010). Finally, decreased dopamine transmission in the prefrontal cortex (PFC) has been observed in abstinent alcohol‐dependent individuals (Narendran *et al*. 2014) supporting the suggested impulsivity trait in AUD. Thus, stabilization of dopamine levels with pharmacological treatment and thereby possibly improving impulse control might be one potential treatment target for AUD.

The monoamine stabilizer (−)‐OSU6162 (OSU6162) has the ability to stimulate, suppress or show no effect on dopamine activity, depending on the prevailing dopaminergic tone (Sonesson *et al*. 1994; Carlsson *et al*. 2004). OSU6162 has affinity for dopaminergic D2 receptors and displays partial agonistic effects *in vitro* (Seeman & Guan 2007; Kara *et al*. 2010) but has failed to demonstrate any intrinsic activity *in vivo* (Sonesson *et al*. 1994; Natesan *et al*. 2006). OSU6162's exact mechanism of action is not fully understood. However, it has been suggested to mediate opposite effects on dopamine activity by acting as an antagonist at both D2 auto‐receptors and hetero‐receptors (Carlsson *et al*. 2004). OSU6162 has been shown to be clinically safe with mild side effects in patients with mental fatigue following stroke and brain trauma (Johansson *et al*. 2012). Thus, an advantage of OSU6162 compared with traditional D2 antagonists might be the lack of extrapyramidal side effects (Carlsson & Carlsson 2006).

We have previously identified OSU6162 as a potential medication for AUD using validated rodent models (Steensland *et al*. 2012). Specifically, OSU6162 attenuates voluntary alcohol consumption, operant alcohol self‐administration under a progressive ratio schedule, alcohol withdrawal symptoms and cue‐induced reinstatement of alcohol seeking in rats that had voluntary consumed alcohol for at least 3 months before treatment (Steensland *et al*. 2012). Based on these results and the favorable side effect profile of OSU6162 (Johansson *et al*. 2012), we recently conducted a proof‐of‐concept double‐blind placebo‐controlled human laboratory study evaluating the effect of OSU6162 on cue‐induced and priming‐induced craving in alcohol‐dependent patients (Khemiri *et al*. 2015). The result showed that OSU6162 attenuated craving after intake of alcohol and induced lower subjective ‘liking’ of the consumed alcohol (Khemiri *et al*. 2015). It is noteworthy that the OSU6162‐induced reduction of craving was only observed in individuals with high level of baseline impulsivity.

In the present study, we further elucidated OSU6162's potential as a novel treatment for AUD by investigating the compound's effects on relapse and impulsive behavior in rats using the alcohol deprivation model (Spanagel 2000) and the five‐choice serial reaction time task (5CSRTT) (Robbins 2002), respectively. In the alcohol deprivation model, renewed access to alcohol solutions after a period of forced abstinence leads to a pronounced, although temporary, increase in alcohol intake in rodents. This phenomenon is referred to as the alcohol deprivation effect (ADE) (Spanagel 2000). The 5CSRTT focus on a specific form of motor impulsivity that is indexed as premature responding (Robbins 2002). An impaired performance in the 5CSRTT following long‐term alcohol exposure has previously been shown in rodents (Walker *et al*. 2011; Irimia *et al*. 2015). Here, we hypothesized that OSU6162 has the ability to attenuate relapse‐like alcohol drinking and improve impulsive control, possibly via a stabilizing action on dopamine activity.

## Materials and Methods

### Animals and housing

Male Rcc Wistar Han Rats (Harlan, Netherlands) weighing approximately 170 g were given at least 1 week to acclimate to the colony room. Rats in the ADE experiment (*n* = 22) were individually housed in plastic Macrolone III cages covered with filter tops (Tecniplast, Italy) on a regular 12‐hours light/dark cycle (lights on at 7 am) with free access to food and water. Rats in the 5CSRTT experiment (*n* = 31) were housed four per cage during the 5CSRTT training and individually from the start of the alcohol drinking phase. The rats in the 5CSRTT experiment were maintained on a reversed 12‐hours light/dark cycle (lights off at 10 am) and food restricted (~85 percent of their free‐feeding weight) throughout the experiment. The study was performed in accordance with the Swedish Animal Welfare Act and approved by the Swedish Ethical Committee on Animal Research in Stockholm (Dnr N475/12 and Dnr N163/14).

### Drugs

The monoamine stabilizer (−)‐OSU6162 was dissolved in 0.9 percent saline, and given subcutaneously at a volume of 5 ml/kg, 60 minutes prior to the start of the drinking session at the ADE test and at the 5CSRTT sessions. The OSU6162 doses were based on our previous results showing that OSU6162 (15 and 30 mg/kg) attenuated several alcohol‐mediated behaviors in rats with no general motor impairments (Steensland *et al*. 2012). Moreover, this dose range induces high striatal D2 receptor occupancy without inducing catalepsy in rats (Natesan *et al*. 2006).

### Intermittent access 20 percent ethanol method

In the intermittent access 20 percent ethanol (IA20E) model, rats had access to alcohol during three 24‐hour drinking sessions per week (Monday, Wednesday and Friday) as described previously (Simms *et al*. 2008; Steensland *et al*. 2012). Water was always available, and no sucrose fading or initiation procedures were needed. Bottles were weighed after 4 and 24 hours of drinking in the ADE experiment and after 24 hours of drinking in the 5CSRTT experiment. Alcohol intake per kilogram of body weight (g/kg), the preference for alcohol over water (the ratio of alcohol to total fluid intake), water intake and total fluid intake were calculated.

### Exp. 1. Effects of OSU6162 on the alcohol deprivation effect in rats

#### The alcohol deprivation method

The alcohol deprivation model (Spanagel 2000) is a relapse‐like drinking model and is based on the observation of a temporary rise in alcohol intake following a period of forced abstinence, a so‐called ADE.

#### The experimental design

**Figure 1:**
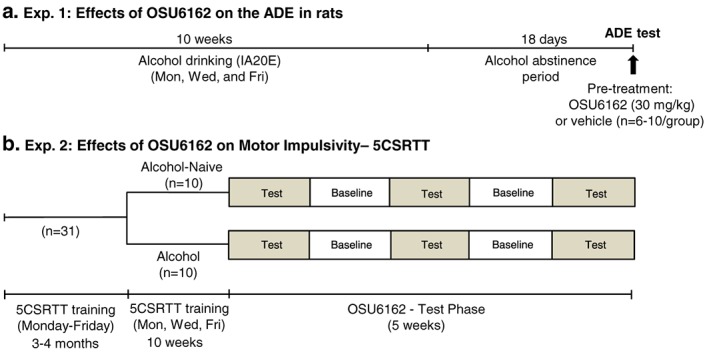
Outline of the experimental timeline for the alcohol deprivation effect (ADE) experiment (a) and for the five‐choice serial reaction time task (5CSRTT) experiment (b). In the ADE experiment (a), rats got voluntary access to alcohol [intermittent access 20 percent ethanol (IA20E; Mon, Wed and Fri)] in their home‐cage during approximately 10 weeks and were thereafter subjected to 18 days of forced alcohol abstinence. Prior to the reintroduction of the alcohol, the rats were divided into two groups with equal baseline alcohol consumption (based on the alcohol intake during the last drinking session before the start of the abstinence period) and given an injection of vehicle (*n* = 10) or OSU6162 (30 mg/kg, subcutaneously, *n* = 6), respectively. In the 5CSRTT experiment (b), food‐restricted rats (*n* = 31) were first trained in the 5CSRTT for 3–4 months. Thereafter, the second phase of the 5CSRTT training began, and stably, responding rats were divided into one alcohol drinking (IA20E; *n* = 10) and one alcohol‐naïve group (*n* = 10). During these 10 weeks, the 5CSRTT was conducted during the mornings of Mon, Wed and Fri, and rats in the alcohol drinking group were given IA20E when the 5CSRTT session was completed. Thereafter, OSU6162 testing was initiated and conducted every other week: 2 days per week during each week (an intertrial interval of 5 s during Tuesdays and an intertrial interval of 7 s during Fridays). The tests were conducted in a counterbalanced order using a Latin square design over a period of 5 weeks. Thus, each rat received all the OSU6162 doses (15 and 30 mg/kg, subcutaneously) and vehicle and served as their own control. The rats were not given access to any alcohol during the test weeks but had regular IA20E during the baseline weeks between the test weeks that consisted of regular 5CSRTT training (Mon, Wed and Fri)

The ADE‐experiment was carried out as described previously (Fredriksson *et al*. 2015). The timeline of Exp. 1 is provided in Figure 1a. Rats (*n* = 22) were given IA20E for approximately 10 weeks and thereafter subjected to 18 days of forced abstinence. Rats consuming over 4.5 g/kg/24 hours were excluded from the experiment (*n* = 5) based on our previous study showing that only rats consuming moderate amounts of alcohol displayed a robust ADE (Fredriksson *et al*. 2015). Rats that did not established a relevant alcohol intake over 10 weeks of drinking were also excluded (*n* = 1). The remaining rats (*n* = 16) were divided into two groups based on their alcohol intake (g/kg/day) during the last alcohol‐drinking session before abstinence and administered either OSU6162 (30 mg/kg) or vehicle (saline), before the reintroduction of the alcohol. The potential of OSU6162 to prevent the ADE was determined by measuring alcohol intake, preference for alcohol over water and water intake 4 and 24 hours after the bottles were reintroduced and compared these values to the corresponding baseline levels before the abstinence period.

### Exp. 2. Effects of OSU6162 on motor impulsivity in alcohol‐naïve and alcohol drinking rats

#### Five‐choice serial reaction time task

The 5CSRTT measures motor impulsivity and attention (Robbins 2002) and was conducted in standard operant conditioning chambers (Med Associate Inc., Georgia, VT, USA) with a pellet dispenser on the right wall and five nose‐poke holes on the left wall. Stimulus, pellet delivery and operant responses were controlled and recorded by Med‐PC IV software (Med Associate Inc.).

The 5CSRTT procedure was performed as described previously (Bari, Dalley, & Robbins 2008), except that a modified training protocol was used (Table 1). Briefly, the start of the session was signaled by the onset of the house light. Rats were trained to nose‐poke in response to a visual stimulus presented randomly in one of the five nose‐poke holes. The duration of the stimulus was initially 20 seconds (Stage 1) and gradually decreased to 1 second (Stage 6) according to a standard training criterion where >80 percent accuracy and <20 percent omissions were required before moving up to the next stage. A correct response (response during stimulus presentation or within the additional limited hold period of 20–5 seconds) was rewarded with a single 45‐mg palatable food pellet (TestDiet, Catalog # 1811155, 12.7 percent fat, 66.7 percent carbohydrate and 20.6 percent protein), delivered in the pellet dispenser on the opposite wall. At the same time, a stimulus light was illuminated to reinforce availability of the reward. No reward was given upon an incorrect response (i.e. response in a non‐illuminated hole) and an omission (i.e. no response when starting a trial). Responding before presentation of the visual stimulus [i.e. within the intertrial interval (ITI)] was recorded as a premature response and restarted the trial without reward delivery. Premature responses were used as a measurement of motor impulsive behavior (Robbins 2002). Incorrect responses, omissions and premature responses were followed by a 5‐second time‐out period. Each session was terminated after 100 trials or 40 minutes, whichever occurred first. Incorrect responses, omissions, premature responses, percentage of correct responses, the total number of trials during a session, latency to respond (i.e. time between stimulus onset and nose poke) and latency to collect the reward (i.e. time to collect the pellet followed a correct response) were recorded.

**Table 1:** Training protocol for the five‐choice serial reaction time task.

*Training stage*	*Stimulation duration*	*Limited hold*	*Intertrial interval*
1	20	20	2
2	10	10	2
3	5	10	5
4	3	5	5
5	1.5	5	5
6	1	5	5

All values are presented in seconds.

#### The experimental design

Overview: The experiment was conducted in food‐restricted rats and consisted of three phases: (1) 5CSRTT training (3–4 months), (2) home‐cage alcohol drinking (10 weeks) and (3) OSU6162–5CSRTT tests (Fig. 1b). Rats were trained in the 5CSRTT, 5 days a week throughout the initial training phase. To maintain a stable baseline 5CSRTT responding during the alcohol drinking phase, rats underwent 5CSRTT training 3 days a week (Monday, Wednesday and Friday). In order to minimize the direct influence of the alcohol consumption (e.g. its caloric value) on the responding in the 5CSRTT, the 5CSRTT training was conducted during the mornings of Mondays, Wednesdays and Fridays (instead of daily Monday–Friday), and the rats in the alcohol drinking group were given access to alcohol first after the completion of the 5CSRTT sessions. Following 10 weeks of IA20E (or only water access), the OSU6162–5CSRTT testing was conducted every other week over a period of 5 weeks. The rats were not given access to any alcohol during the test weeks. However, to make sure that the rats would still be exposed to alcohol throughout the experiment, they were given regular alcohol access during the in‐between baseline weeks with regular 5CSRTT training (Fig. 1b). The handling, training and the experimental parameters were identical for groups of alcohol‐naïve and alcohol‐exposed rats. However, alcohol‐naïve control rats were exposed only to water during the second and third phase of the experiment.

When the rats had been drinking alcohol (*n* = 10) or water (*n* = 10) for 10 weeks, the OSU6162–5CSRTT testing began. All OSU6162 doses (15 or 30 mg/kg, subcutaneously) and vehicle were given to all rats in a counterbalanced order. OSU6162 tests were conducted twice a week (Tuesdays and Fridays) every other week, with baseline 5CSRTT training sessions on the intervening days. On Tuesdays, the rats were tested under an ITI of 5 seconds, i.e. the same ITI length used on baseline training. In contrast, on Fridays, the rats were challenged with a prolonged waiting period before presentation of the visual stimulus (i.e. ITI were changed from 5 to 7 seconds). This manipulation has previously been shown to provoke premature responses (Dalley *et al*. 2007). The number of premature responses, trials, correct responses, incorrect responses, omissions, latency to collect and latency to respond were recorded and compared between OSU6162 and vehicle.

#### Statistics

Statistical analysis of the ADE experiment was performed using paired Student's *t*‐test (GraphPad Prism, San Diego, CA, USA). It was priori determined to compare difference before and after abstinence period within each treatment group. The 5CSRTT data were analyzed using two‐way repeated‐measures ANOVA [IBM SPSS Statistics 20 (IBM)], with the within‐subject factors of treatment (baseline, 0, 15 and 30 mg/kg) and condition (ITI5s and ITI7s session). Baseline was defined as mean of responding during the week before the first OSU6162–5CSRTT testing. It was priori determined that significant main effects together with interaction effects (*P* < 0.05) within each condition should be followed up with one‐way repeated‐measures ANOVA. Greenhouse–Geisser adjustments were used when sphericity assumption was violated. For the 5CSRTT experiment, 11 rats (of 31 in total) were excluded due to failure to reach stage 6 (*n* = 4), instable baseline on stage 6 (*n* = 2) or outliers during any test session (*n* = 5).

## Results

### Exp. 1. OSU6162 treatment blunted the alcohol deprivation effect in rats

The effects of acute OSU6162 (30 mg/kg) and vehicle (saline) treatment on the ADE were evaluated in a group of rats that voluntarily had consumed alcohol for approximately 10 weeks in the IA20E model before the forced abstinence period (18 days). Based on the alcohol intake during the last drinking day before the abstinence period, rats were divided in two groups with equal alcohol consumption (OSU6162: 3.3 ± 0.3 g/kg/24 hours; vehicle: 3.2 ± 0.2 g/kg/24 hours). Overall, OSU6162 treatment blunted the ADE. The statistical results for the different measurements are provided in the succeeding texts.

**Figure 2:**
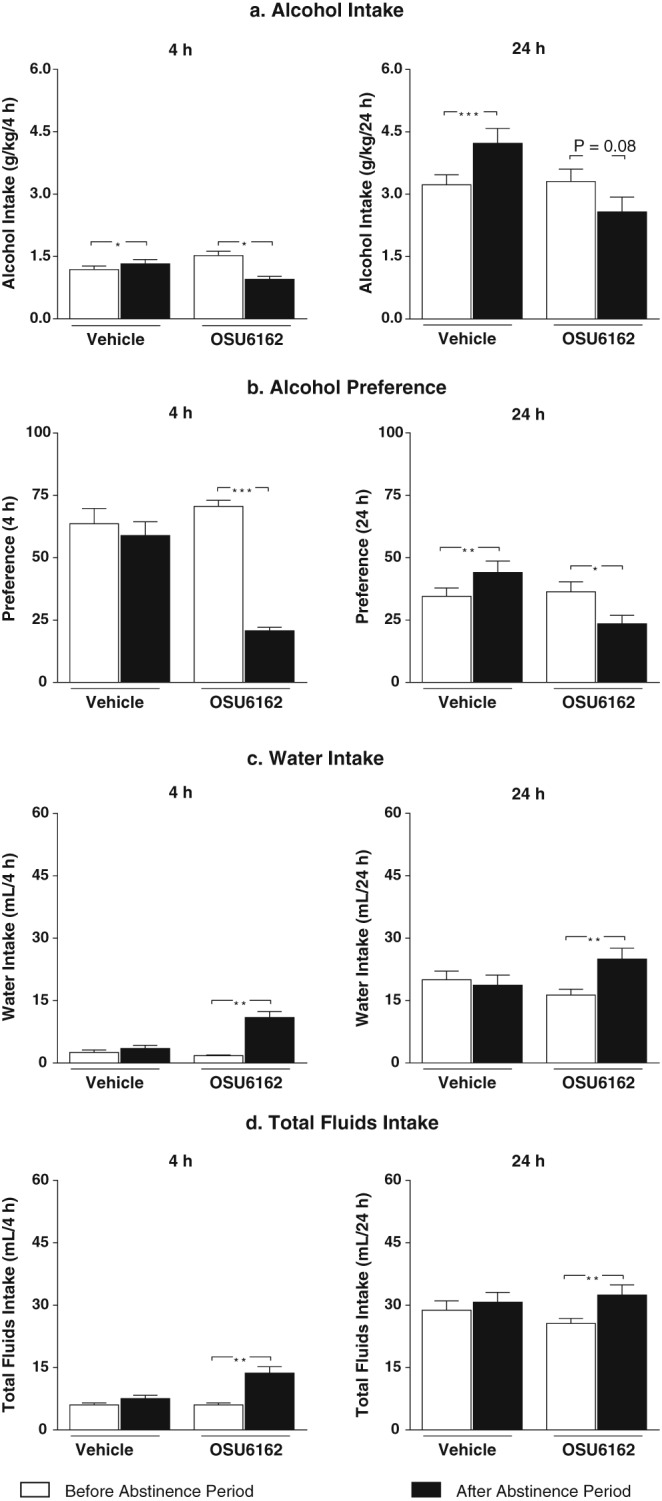
Rats got voluntary intermittent access to alcohol in their home‐cage during approximately 10 weeks and were thereafter subjected to 18 days of forced alcohol abstinence. Prior to the reintroduction of the alcohol, the rats were divided into two groups with equal baseline alcohol consumption (OSU6162 = 3.3 ± 0.3 g/kg/24 hours; vehicle = 3.2 ± 0.2 g/kg/24 hours) and given an injection of vehicle (*n* = 10) or OSU6162 (30 mg/kg; *n* = 6). An alcohol deprivation effect was observed in vehicle‐treated rats (a: 4 hours: left panel; 24 hours: right panel), whereas OSU6162 treatment significantly attenuated the alcohol deprivation effect as shown by a decreased alcohol intake at the 4‐hour timepoint (a: left panel) and a non‐significant trend towards a reduction in alcohol intake at the 24‐hour timepoint (a: right panel). No difference was observed in water intake (c: 4 hours: left panel; 24 hours: right panel) or total fluid intake (d: 4 hours: left panel; 24 hours: right panel) in the vehicle‐treated rats. Moreover, in rats treated with vehicle, a significant increased preference for alcohol was seen at the 24‐hour timepoint (b: 4 hours: left panel; 24 hours: right panel). In contrast, the water intake (c: 4 hours: left panel; 24 hours: right panel) and the total fluid intake (d: 4 hours: left panel; 24 hours: right panel) were significantly increased in rats treated with OSU6162, and consequently, the preference for alcohol was significantly reduced in these rats (b: 4 hours: left panel; 24 hours: right panel) compared with baseline. All values are expressed as mean ± standard error of the mean, ^*^ *P* < 0.05; ^**^ *P* < 0.01 ^***^ *P* < 0.01; compared with corresponding baseline (paired Student's *t*‐test within each treatment group)

Following the reintroduction of the alcohol after the period of abstinence, the vehicle‐treated rats showed a significantly increased alcohol consumption compared with corresponding baseline for both timepoints (Fig. 2a: 4 hours: *P* < 0.05, left panel; 24 hours: *P* < 0.001, right panel). In contrast, in rats treated with OSU6162, a significantly decreased alcohol intake was observed after 4 hours of alcohol drinking (4 hours: *P* < 0.05, Fig. 2a left panel). At the 24‐hour timepoint, there was a non‐significant trend to a decreased alcohol intake compared with baseline (24 hours: *P* = 0.08, Fig. 2a right panel). The ADE experiment further revealed a significantly increased preference for the alcohol over water after the abstinence period compared with baseline for vehicle‐treated rats at the 24‐hour (*P* < 0.01, Fig. 2b right panel) but not at the 4‐hour timepoint (*P* > 0.05, Fig. 2b left panel). Moreover, the vehicle‐treated rats showed no significant effect on water intake (Fig. 2c: 4 hours: *P* > 0.05, left panel; 24 hours: *P* > 0.05, right panel) or total fluid intake (Fig. 2d: 4 hours: *P* > 0.05, left panel; 24 hours: *P* > 0.05, right panel) compared with corresponding baseline at any timepoints. In contrast, the OSU6162‐treated rats had a significantly decreased preference for alcohol (Fig. 2b: 4 hours: *P* < 0.0001, left panel; 24 hours: *P* < 0.05, right panel), increased water intake (Fig. 2c: 4 hours: *P* < 0.01, left panel; 24 hours: *P* < 0.01, right panel) and increased total fluid intake (Fig. 2d: 4 hours: *P* < 0.01, left panel; 24 hours: *P* < 0.01, right panel), at both timepoints compared with corresponding baseline.

### Exp. 2. OSU6162 treatment improve motor impulsive behavior in rats

The effect of acute OSU6162 treatment on motor impulsivity was evaluated in rats that had voluntarily consumed alcohol or water for 10 weeks before the 5CSRTT test. For the alcohol drinking rats, the mean alcohol intake during the last week of drinking was 2.1 ± 0.38 g/kg/24 hours. Overall, OSU6162 treatment blunted the increased premature responding induced by a prolonged ITI in both alcohol and alcohol‐naïve rats. The statistical results for the different measurements are provided in the succeeding texts.

### Alcohol drinking rats

#### Premature responses (Fig. 3a)

**Figure 3:**
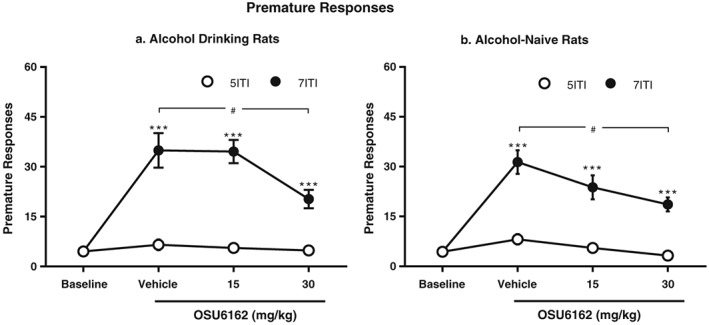
Following 3–4 months of five‐choice serial reaction time task training and 10 weeks of home‐cage alcohol or water drinking, using the intermittent access 20 percent ethanol method, both alcohol (a) and alcohol‐naïve rats (b) significantly increased their premature responses compared with baseline when the intertrial interval (ITI) was prolonged from 5 to 7 seconds in the five‐choice serial reaction time task. However, the highest OSU6162 (30 mg/kg) dose significantly reduced the number of premature responses compared with vehicle in both alcohol (a) and water (b) pre‐exposed rats. All values are presented as mean ± standard error of the mean, *n* = 10 per group; ^***^ *P* < 0.001 compared with corresponding baseline and ^#^ *P* < 0.05 compared with corresponding vehicle within the ITI7s session

The analyses of premature responses showed an overall main effect on condition (ITI5s or ITI7s session) [*F*(1,9) = 158.0, *P* < 0.001], treatment [*F*(3,27) = 15.5, *P* < 0.001] and a significant interaction between the two factor [*F*(3,27) = 17.3, *P* < 0.001]. *Post hoc* analysis revealed that the prolonged waiting period (ITI7s) significantly increased premature responses compared with baseline. Furthermore, the highest OSU6162 dose (30 mg/kg) significantly decreased premature responses compared with vehicle. There were no significant *post hoc* effects during the ITI5s sessions (baseline condition).

#### Number of trials (Fig. 4a)

**Figure 4:**
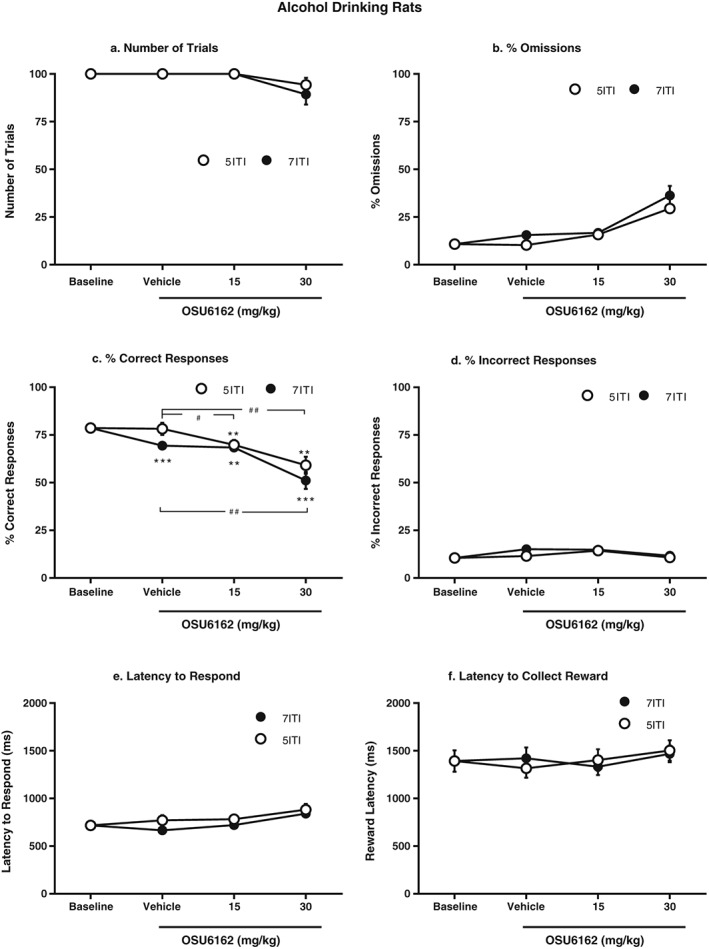
Following 3–4 months of five‐choice serial reaction time task (5CSRTT) training and 10 weeks of home‐cage alcohol drinking, using the intermittent access 20 percent ethanol method, OSU6162 (30 mg/kg) pre‐treatment significantly decreased the percentage of correct responses compared with baseline and vehicle when the intertrial interval (ITI) was prolonged from 5 to 7 seconds in the 5CSRTT (c). Moreover, vehicle and OSU6162 (15 mg/kg) pre‐treatment significantly decreased the percentage of correct responses compared with baseline during the ITI7s session (c). In addition, both OSU6162 doses significantly decreased the percentage of correct responses compared with baseline and vehicle for the ITI5s session (c). OSU6162 pre‐treatment had no significant effect on the number of trials (a), omission rate (b), percentage of incorrect responses (d), latency to respond (e) or latency to collect the reward (f) for either the ITI5s session or the ITI7s session. All values are presented as mean ± standard error of the mean, *n* = 10; ^**^ *P* < 0.01, ^***^ *P* < 0.001 compared with corresponding baseline within the ITI5s or ITI7s session and ^#^ *P* < 0.05, ^##^ *P* < 0.01 compared with corresponding vehicle within the ITI5s or ITI7s session

The analyses of the number of trials showed an overall main effect on treatment [*F*(3,27) = 4.6, *P* = 0.01], but no main effect on condition [*F*(1,9) = 1.0, ns] and no significant interaction between the two factors [*F*(3,27) = 1.0, ns].

#### Omission (Fig. 4b)

The analyses of the omission rate showed an overall main effect on condition [*F*(1,9) = 7.1, *P* < 0.05] and treatment [*F*(1.3,11.7) = 13.2, *P* < 0.01], but no significant interaction between the two factors [*F*(3,27) = 2.9, ns].

#### Correct responses (Fig. 4c)

The analyses of the percentage of correct responses showed an overall main effect on condition [*F*(1,9) = 12.6, *P* < 0.01], treatment [*F*(1.4,12.5) = 16.4, *P* < 0.01] and a significant interaction between the two factors [*F*(3,27) = 4.6, *P* = 0.01]. *Post hoc* analysis further revealed that both OSU6162 doses (15 and 30 mg/kg) significantly decreased the percentage of correct responses compared with both vehicle and baseline during the ITI5s sessions. Moreover, vehicle and the lower OSU6162 dose (15 mg/kg) significantly decreased the percentage of correct responses compared with baseline during the ITI7s sessions. In addition, the higher OSU6162 dose (30 mg/kg) significantly decreased the percentage of correct responses compared with all treatments during the ITI7s sessions.

#### Incorrect responses (Fig. 4d)

The analyses of the percentage of incorrect responses showed an overall main effect on treatment [*F*(3,27) = 4.0, *P* < 0.05], but no main effect on condition [*F*(1,9) = 1.7, ns] or a significant interaction between the two factors [*F*(3,27) = 1.6, ns].

#### Latency to respond (Fig. 4e)

The analyses of the latency to respond showed an overall main effect on condition [*F*(1,9) = 6.8, *P* < 0.05] and treatment [*F*(3,27) = 13.7, *P* < 0.001] but no significant interaction between the two factors [*F*(1.5,13.9) = 1.2, ns].

#### Latency to collect the reward (Fig. 4f)

The analyses of the latency to collect the reward showed no overall main effect on condition [*F*(1,9) = 0.0, ns] or treatment [*F*(3.27) = 2.4, ns] as well as no significant interaction between the two factors [*F*(3,27) = 1.0, ns].

### Alcohol‐naïve rats

#### Premature responses (Fig. 3b)

The analyses of premature responses showed an overall main effect on condition (ITI5s or ITI7s session) [*F*(1,9) = 259.7, *P* < 0.001], treatment [*F*(3,27) = 15.0, *P* < 0.001] and a significant interaction between the two factors [*F*(1.8,15.9) = 15.2, *P* < 0.001]. *Post hoc* analysis revealed that the prolonged waiting period (ITI7s) significantly increased premature responses, compared with baseline. Furthermore, the highest OSU6162 dose (30 mg/kg) significantly decreased premature responses compared with vehicle. There were no significant *post hoc* effects during the ITI5s sessions (baseline condition).

#### Number of trials (Fig. 5a)

**Figure 5:**
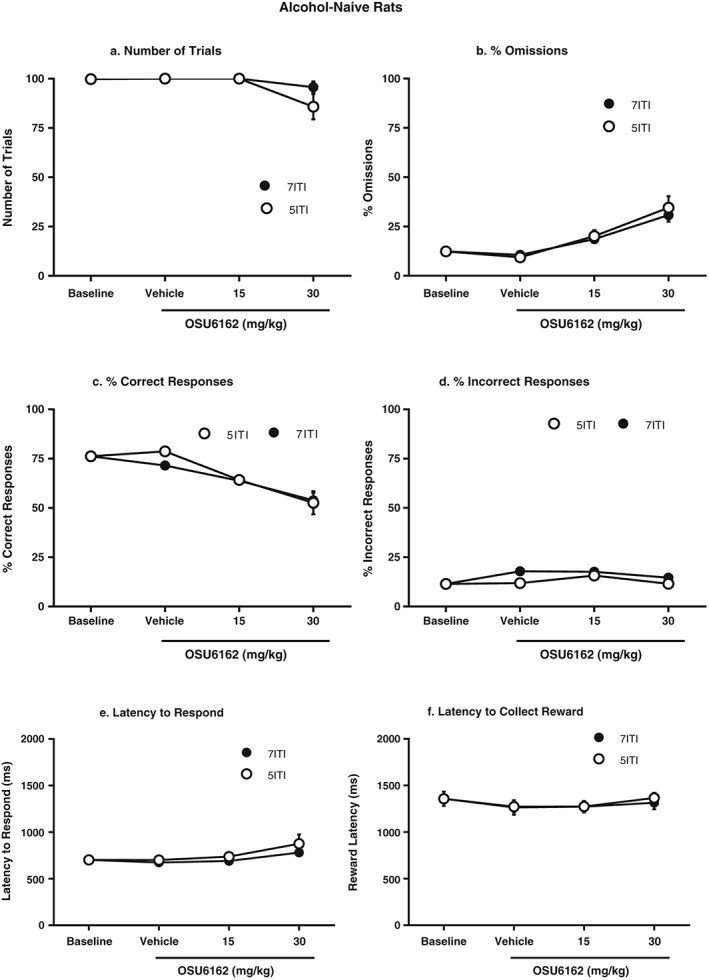
Following 3–4 months of five‐choice serial reaction time task training and 10 weeks of home‐cage water drinking, OSU6162 pre‐treatment had no significant effect on number of trials (a), omission rate (b), percentage of correct responses (c), percentage of incorrect responses (d), latency to respond (e) or latency to collect the reward (f) for either the intertrial interval (ITI) 5s session or the ITI7s session. All values are presented as mean ± standard error of the mean, *n* = 10

The analyses of the number of trials showed an overall main effect on treatment [*F*(3,27) = 6.6, *P* < 0.01], but no main effect on condition [*F*(1,9) = 2.0, ns] or a significant interaction between the two factors [*F*(3,27) = 2.0, ns].

#### Omission (Fig. 5b)

The analyses of the omission rate showed an overall main effect on treatment [*F*(1.5,13.7) = 25.5, *P* < 0.001], but no main effect on condition [*F*(1,9) = 0.3, ns] or a significant interaction between the two factors [*F*(1.3,11.9) = 0.3, ns].

#### Correct responses (Fig. 5c)

The analyses of the percentage of correct responses showed an overall main effect on treatment [*F*(1.4,12.3) = 27.2, *P* < 0.001], but no main effect on condition [*F*(1,9) = 1.2, ns] or a significant interaction between the two factors [*F*(1.3,12.0) = 1.1, ns].

#### Incorrect responses (Fig. 5d)

The analyses of the percentage of incorrect responses showed an overall main effect on condition [*F*(1,9) = 13.5, *P* < 0.01] and treatment [*F*(3,27) = 7.1, *P* = 0.001], but no significant interaction between the two factors [*F*(1.5,13.9) = 2.4, ns].

#### Latency to respond (Fig. 5e)

The analyses of the latency to respond showed no overall main effect on condition [*F*(1,9) = 2.0, ns], treatment [*F*(1.4,12.7) = 3.8, ns] or a significant interaction between the two factors [*F*(1.4,13.0) = 0.7, ns].

#### Latency to collect the reward (Fig. 5f)

The analyses of the latency to collect the reward showed no overall main effect on condition [*F*(1,9) = 0.9, ns], an overall main effect of treatment [*F*(1.6,14.2) = 4.2, *P* < 0.05], but no significant interaction between the two factors [*F*(3.27) = 0.4, ns].

## Discussion

The present study shows for the first time that the monoamine stabilizer OSU6162 blunted the ADE in long‐term drinking rats and improved impulse control in both alcohol‐naïve and alcohol drinking rats. These results give further support for the potential of OSU6162 as a novel AUD medication. Together with our recent study showing that OSU6162's ability to attenuate alcohol‐induced craving in alcohol dependent individuals was driven by those with high baseline impulsivity (Khemiri *et al*. 2015), the present results indicate that improvement of motor impulse control might be one mechanism behind OSU6162's ability to attenuate alcohol‐mediated behaviors (Steensland *et al*. 2012).

In the present study, an ADE was observed in the vehicle‐treated rats, whereas OSU6162 treatment prevented such increase in alcohol intake. The increased demand for alcohol in the alcohol deprivation model is suggested to be clearly dissociated from normal eating or drinking behavior (Sanchis‐Segura & Spanagel 2006) and might therefore resembles a typical lapse or relapse situation in AUD patients. Thus, the present ADE experiment complements our previous results showing that OSU6162 attenuates cue/priming‐induced reinstatement (Steensland *et al*. 2012). The present results together with studies showing that naltrexone and acamprosate [two currently Food and Drug Administration (FDA)‐approved AUD medications] prevent the ADE in rats (Spanagel & Zieglgansberger 1997; Heyser *et al*. 2003; Fredriksson *et al*. 2015), and craving‐induced relapse in humans (Soyka & Rosner 2008), indicate that OSU6162 might have potential to prevent relapse‐like drinking behavior also in a clinical situation. Moreover, in the ADE experiment, the OSU6162‐treated rats showed a significant decrease in the preference for alcohol. The vehicle‐treated rats, on the other hand, showed an increased alcohol preference. These results demonstrate that OSU6162‐treated rats in greater extent prefer drinking water instead of alcohol. These results correlate well with our previous results showing that OSU6162 decreased alcohol intake and alcohol preference in the IA20E but had no significant effect on water intake on water days or intake of a salty solution (Steensland *et al*. 2012).

The mechanism behind OSU6162's ability to attenuate an ADE is not fully understood. The ADE has been suggested to be interpreted as loss of control with regard to the level of use or the termination of alcohol intake (Vengeliene *et al*. 2009). The exact mechanism behind the induction of an ADE is not clear, but it has been suggested that alcohol itself might act as a cue (i.e. smell) or a priming stimuli triggering the ADE (Spanagel 2000). Furthermore, during protracted abstinence, low synaptic dopamine levels might contribute to craving and relapse to alcohol abuse (Guardia *et al*. 2000). In fact, rats undergoing alcohol withdrawal will self‐administer just enough alcohol to return dopamine levels to normal (Weiss *et al*. 1996). Thus, OSU6162 might restore an alcohol‐induced dopamine deficiency and thereby attenuating the craving for alcohol, with a decrease in relapse‐like drinking behavior as a result. This suggestion is supported by our recent microdialysis study (Feltmann *et al*. 2016) showing that long‐term voluntary alcohol drinking in the IA20E model leads to a significantly reduced dopamine output in the NAcc compared with alcohol‐naïve rats. Moreover, an alcohol‐induced dopamine peak was blunted, and there was a subsequent shift in dopamine levels below baseline in the long‐term drinking, compared with the alcohol‐naïve rats. Interestingly, in the alcohol‐exposed rats, OSU6162 pre‐treatment normalized the alcohol‐induced dopamine peak and prevented the dopamine levels to dip below baseline (Feltmann *et al*. 2016). However, it should be noted that the experimental conditions in our previous microdialysis study were not fully comparable with those in the present study. For example, the microdialysis was performed in rats undergoing acute (24 hours) abstinence, whereas the rats in the present ADE experiment underwent protracted (18 days) abstinence. Thus, further studies are needed to elucidate the effects of OSU6162 on the dopamine output in long‐term drinking rats undergoing protracted abstinence.

In the present 5CSRTT experiment, both alcohol and alcohol‐naïve rats significantly increased their premature responses, from an equal baseline level, when challenged with a prolonged waiting period (i.e. when the ITI was prolonged from 5 to 7 seconds), confirming that the manipulation provoked motor impulsive behavior. The highest dose of OSU6162 (30 mg/kg) improved motor impulsivity, as shown by a significantly decreased premature responding during the 7‐second ITI compared with vehicle treatment. These results indicate that OSU6162 might have beneficial effects on impulse control ‐ a characteristic desirable for a potential alcohol dependent medication because alcohol dependent individuals often demonstrate impaired impulse control (Bjork *et al*. 2004; Kamarajan *et al*. 2005; Lawrence *et al*. 2009; Voon *et al*. 2014).

There are some potential limitations of the 5CSRTT experiment that should be discussed. First, as noted in the result Results section, the OSU6162 treatment was associated with a modest increase in some of the latencies and omission rates together with a decrease in correct responding. In this context, it could possibly be argued that OSU6162 induced a motor slowing or sedating effect that in turn could contribute to the attenuation of premature response. However, the increase in latencies was quite modest. Even following treatment with the highest OSU6162 dose (30 mg/kg), the rats responded within 841 ± 42 and 882 ± 60 milliseconds during the ITI5s and ITI7s session, respectively. This fast responding clearly reflects an intact general motor activity. The lack of sedative effect is further supported by previous studies showing that OSU6162, using the same dose range as in the present study, had no effect on general motor activity (Sonesson *et al*. 1994; Natesan *et al*. 2006; Steensland *et al*. 2012; Studer *et al*. 2016). Moreover, Benaliouad *et al*. (2009) have shown, using electrical brain stimulation, that OSU6162 (30–60 mg/kg) treatment produced a dose‐dependent reduction of reward with no change in the capacity of the animals to produce the operant response. Thus, an OSU6162‐induced sedating effect in the rats in the present study seems unlikely. Second, the decreased number of trials following the treatment with the highest OSU6162 (30 mg/kg) dose could indicate a decreased motivation to seek the palatable pellets that was given as a reward during the 5CSRTT sessions. Given the ability of OSU6162 to attenuate, not only voluntary alcohol consumption but also sucrose consumption (Steensland *et al*. 2012) as well as binge eating and seeking of sucrose pellets (Feltmann *et al*. 2018), this suggestion is quite likely. Nevertheless, a decreased motivation to seek the pellets should have less impact on the OSU6162‐induced reduction in the number of premature responses when the rats in fact were carrying out as many as 85–95 of the 100 trials during a session. Thus, we believe that the present 5CSRTT results indicate that OSU6162 has the potential to attenuate both impulsivity and intake of palatable foods. Reduced appetite is indeed a common side effect from all FDA‐approved attention deficit hyperactivity disorder medications, including lisdexamfetamine, methylphenidate and atomoxetine (Clavenna & Bonati 2017). In particular, lisdexamfetamine is FDA‐approved for both attention deficit hyperactivity disorder and binge‐eating disorder (Davis & Attia 2017). Thus, the present results, together with our recent study showing that OSU6162 blunts binge‐like eating in rats (Feltmann *et al*. 2018), indicate that OSU6162 might have a similar profile as lisdexamfetamine.

The dopamine system and the D2 receptors in particular are suggested to be involved in both AUD (Tupala & Tiihonen 2004) and the complex regulation of impulsive behavior (Dalley & Roiser 2012). In alcohol dependent individuals, a decreased dopamine transmission in PFC (Narendran *et al*. 2014) has been associated with a reduction in striatal D2/D3 receptors and a decreased metabolic activity in prefrontal regions necessary for executive control (e.g. inhibitory control) (Volkow *et al*. 2017). In addition, low striatal D2 receptor availability has been linked to increased impulsivity in both social drinkers and individuals with AUD (Oberlin *et al*. 2015). Thus, it is possible that OSU6162 might normalize abnormal dopamine activity by acting at D2 receptors in the striatum and thereby strengthen the function of cortico‐striatal circuitries, which potentially might lead to an improved impulse control. In fact, striatal dopamine receptors have been suggested to play an important role in the specific type of impulsivity measured with 5CSRTT (Dalley & Roiser 2012). In a recent study, low doses of quinpirole (a D2/D3 agonist) and sumanirole (a D2 agonist) selectively reduced impulsivity on the 5CSRTT (Fernando *et al*. 2012). Moreover, administration of the quinpirole into the orbitofrontal cortex produced a generalized disruption in 5CSRTT performance, involving premature responding, accuracy, omissions and response latencies (Winstanley *et al*. 2010). In addition, intra‐NAcc administration of D2/D3 antagonist completely blocked the increased effect of amphetamine on premature responding (Cole & Robbins 1987; Pattij *et al*. 2007). Furthermore, intra‐NAcc core administration of sulpiride (a D2/D3 antagonist) attenuated the increased premature responding induced by selective PFC lesions (Pezze, Dalley, & Robbins 2009). In contrast, intra‐NAcc core administration of a D2 antagonist had no effect on inhibitory control by itself (van Gaalen *et al*. 2006; Pattij *et al*. 2007). Taken together, these results highlight an important role of dopamine with inputs to the NAcc, presumably the core subregion, as an important substrate in the regulation of impulsive behavior in the 5CSRTT. OSU6162's potential to target the dopamine system in brain regions relevant for 5CSRTT is supported by a recent human positron emission tomography study showing that OSU6162 preferentially binds to D2/D3 receptors in the striatum (Tolboom *et al*. 2015).

In conclusion, the present study shows for the first time that OSU6162 has the ability to attenuate an ADE and improve motor impulsivity in rats. These results provide further support for OSU6162 as a potential novel treatment for AUD. Indeed, several clinical studies demonstrate an impaired impulse control in AUD individuals (Bjork *et al*. 2004; Kamarajan *et al*. 2005; Lawrence *et al*. 2009; Voon *et al*. 2014), which highlights motor impulsivity as a potential treatment target during AUD. This suggestion is supported by our human laboratory study showing that OSU6162 attenuates priming‐induced craving only in alcohol dependent individuals with high baseline impulsivity (Khemiri *et al*. 2015). We therefore hypothesize that OSU6162's ability to improve impulse control might help AUD individuals to over‐ride a compulsive drug‐taking behavior in response to craving and thereby possibly prevent relapse to alcohol drinking.
